# Obstetrics and Diseases of Women and Children

**Published:** 1858-09

**Authors:** 


### OBSTETRICS, AND DISEASES OF WOMEN AND CHILDREN.

### IV. *On the Pathogeny of Hydrorrhœa of Pregnant Women.* By PROF. C. BRAUN.

HYDRORRHŒA, as is well known, is a periodic discharge of a yellowish, sero-albuminous fluid, which may either occur in drops, or in quantities of even several pints, from the genitals of pregnant women, but has no connection with the rupture of the fœtal membranes, and the escape of the liquor amnii. It is observed, generally, in the latter months of pregnancy, hardly ever before the third month; may occur either once or several times, imitates sometimes even the menstrual period, leads rarely to an interruption of pregnancy, ceases generally with delivery, but may continue in exceptional cases for some time during the puerperal state. On account of the red color of the excretion, the hydrorrhœic fluid is not unrarely mistaken for menstrual blood. Hydrorrhœa was considered a discharge of "false waters," and its symptoms were explained by numerous contradictory hypotheses.

The so-called "false waters" were believed to be either a secretion of the allantois, or to come from the magma reticulé and rupture of the chorion, or from the brownish mucus (hydroperione) existing between the decidua vera and the decidua reflexa, or from a transudation of liquor amnii through the fœtal membranes, or from the rupture of a hydatid situated in the vicinity of the cervix, or, finally, from the rupture of a supernumerary ovum. Some even considered it to be the real liquor amnii, and thought that the false waters were produced by a rupture of both membranes of the ovum taking place at a considerable distance from the os uteri.

Naegele was the first who recognized hydrorrhœa to be a product of secretion of the uterus, but believed that this did not reach the cavity of the ovum, on account of a disturbed endosmotic action of the fœtal membranes. The author has investigated this morbid process more fully, and has described hydrorrhœa in his "Manual of Midwifery," p. 534, as an albuminous exudation of the inner surface of the uterus (endometritis serosa,) which occurs in an intermittent manner, lifts off a part of the chorion from the decidua, or the decidua itself, forming thereby a more or less extensive reservoir, and is discharged at intervals as it reaches the os uteri.

This view is corroborated by the following circumstances:—That similar large albuminous excretions are observed, also, in unimpregnated women, attending several pseudo-plasmata, particularly fibroids of the uterus; that after birth neither chorion nor amnion show any signs of disease; that the quantity of the amniotic liquor is not diminished in consequence of a hydrorrhœa; also that the most plausible theories in regard to the formation of the amniotic liquor are not contradictory to this view; and that *microscopical investigation of a placenta,*

*born after hydrorrhœa, reveals, on its convex surface, a new formation, having the appearance of a tender membrane consisting of areolar tissue,* as is confirmed by the following case:—

On the 7th of June, 1857, a healthy woman, twenty-three years of age, was received in Dr. Braun's clinic. The patient had lost, several times during her pregnancy, and also on the day of her admission, a considerable quantity of water from her genitals. Careful observation during three days convinced the attendants of the regular formation of the bag of waters, of the timely bursting of the same, and of the discharge of about six pounds of amniotic liquor. The child, born apparently dead, was premature, and had upon the palms of its hands and soles of its feet, pustules, (pemphigus neonatorum,) and numerous lentil-shaped spots deprived of epidermis. The after-birth was expelled naturally; the membranes of the ovum were nowhere perforated except at the place where the child had gone through. The child died the next day; the patient remained well, and was dismissed the second week after delivery.

Professor Wedl made a microscopic investigation of the membranes of the ovum, but was unable to discover the smallest trace of an abnormal rupture; on the convex surface of the placenta he found, however, a tender membrane of areolar tissue, which was hanging down here and there in shreds. This circumstance seems to indicate that an exudation took place between the wall of the uterus and the placenta, which would explain, very simply, the discharge of the so-called false waters, and the retention of the new formation of areolar tissue.—(*Zeitschrift der Gesellschaft der Aerzte zu Wien*, 1858. No. 18.)

V. *On Amputation of the Neck of the Uterus in Prolapsus of this Organ.* By DR. C. MAYER.

Dr. Mayer applies this method of treatment to prolapsus of the uterus in consequence of hypertrophy of its neck. His proceeding consists in removing the whole os uteri with the bistoury, after having fixed the uterus by means of hooks. The hemorrhage, which is often considerable, is arrested by the actual cautery; the dressing consists of tampons of charpie which retain the uterus in place; cicatrization of the wound is hastened by cauterizing it from time to time with nitrate of silver. Dr. Mayer prefers the bistoury to the écraseur linéaire, as the latter is apt to injure the neighboring organs. In a case treated in Langenbeck's clinic, where this instrument was used to remove an epithelial cancer of the neck of the uterus, the patient died on the third day, and the autopsy revealed a perforation of the bladder and of the peritoneum.

In three cases reported by the author, where the hypertrophied neck appeared outside the vulva, its amputation sufficed to arrest the very serious symptoms which accompanied the prolapsus, and to diminish considerably the volume of the engorged uterus; there remained only a slight prolapsus of the vagina which could be easily kept reduced by means of a tampon of charpie. A fourth case is recorded in the tenth volume of *Virchow's Archives*.—(*Monatsschr. f. Geburtskunde*, xi. bd., pp. 161–163.)

VI. *On Linear Ecrasement of the Cervix Uteri.* By DR. BRESLAU, of München. (Communication to the gynaekological section of the Thirty-third Congress of German Naturalists and Physicians.)

Dr. Breslau has used the écraseur up to this time, in four cases, for amputation of the neck of the womb, three times on account of carcinoma, and once on account of chronic hypertrophy and induration. In all cases the loss of blood was insignificant, and no secondary hemorrhage took place. In two cases a portion of the anterior wall of the vagina was grasped within the chain of the écraseur on tightening it, and was squeezed off, and once a loop of intestine prolapsed through the orifice thus made. In extirpating the cervix uteri, which is very vascular in its normal state, but becomes much more so when diseased, on account of the enlargement and new formation of blood-vessels, the écraseur is far preferable to the knife and scissors, as in using it hemorrhage is almost entirely avoided. Its employment in carcinoma of the cervix uteri is indicated only when between the diseased tissue and the cul-de-sac of the vagina a healthy place, at least 1‴ in width, can be found. The loop of the instrument must not be put on higher than necessary, because in tightening it the parts in close proximity to it are drawn along from above downward into the loop, whereby injuries may be inflicted upon the neighboring organs. In some cases it would be advantageous to use a curved écraseur, the convex side of which should, however, not be directed toward the sacrum, but toward the symphysis of the pubis.—(*Monatsschr. f. Geburtsk.* xi., Jan. 1858.)

VII. *Treatment of Engorgement of the Cervix Uteri with Ointment of the Chloroiodide of Mercury.* M. CH. BERNARD.

The preparations used by M. Ch. Bernard in the treatment of engorgement of the cervix uteri is the same as that proposed by M. Rochard. The formula for making it, as published in the *Gazette Hebdomadaire,* 1856, p. 172, is the following :—Take one part of iodine and two parts of calomel; reduce the calomel to a coarse powder, and introduce it into a matrass, heat it gently while stirring it, until it commences to sublimate, then add iodine in small portions, and the combination takes place.

For application to the uterus, from fifty to seventy-five centigrammes of this salt are mixed with sixty grammes of fat.

M. Rochard recommended the use of this ointment only in cases of simple and subacute engorgement showing a tendency to become chronic. It has been applied in the following manner :—The neck of the uterus, brought completely into view by means of a trivalve speculum, is cleared of the mucus covering it with charpie or wadding, which we have always found sufficient, or with a tampon soaked in glycerin, and applied the day before to the cervix, as recommended by M. Rochard. On the other hand, a moderately thick pledget of charpie of a little larger dimension than the cervix is prepared, and its centre covered by a thin layer of the ointment, so that its borders remaining dry may defend the vaginal mucous membrane from contact with the remedy which would otherwise occasion inflammation, (M. Rochard.) Then the pledget is carried up to the neck either with a dressing forceps, or, what is better, and has often been used by M. Rochard, by means of a wooden tube, one extremity of which (that upon which the pledget is placed) is proportionate in dimensions to the cervix uteri, and in which slides a stopper which applies the pledget exactly to the organ. This done, the vagina is filled with balls of wadding, and the speculum is withdrawn. Care has to be taken not to cram the vagina, as it would occasion unnecessary inconvenience and pain to the patient. Six or seven hours after the application of the ointment the speculum is again introduced, the different parts of the dressing are removed, and the cervix is laid bare, which is always found covered with an albuminous exudation.

M. Ch. Bernard reports the following cases :—

CASE I.—Voluminous cervix occupying the whole field of the speculum, and presenting some small granulations on the posterior lip ; the engorgement is probably of two years' standing. Application of the ointment on the 21st of September and 1st of October. On the 12th of October the cervix uteri "seems to have returned nearly to its normal dimensions." The functional state of the parts is much ameliorated.

CASE II.—Considerable engorgement of the cervix of fifteen days' duration; a little redness, and some small ulcerations on the os. Only one application on the 7th of September. On the 9th of October "the patient left the hospital, feeling entirely well; she does not complain of pain in the hypogastrium, and the cervix uteri seems to have reassumed its ordinary volume and consistence."

CASE III.—Cervix very hard and much engorged; the disease commenced about two years ago. A single application on the 17th of September. On the 11th of October, "diminution of the volume and hardness of the neck, which seems to be reduced to its normal dimensions. The patient feeling well, demands to be dismissed."

CASE IV.—Old engorgement, which had disappeared after injections of carbonic acid, but has returned in the last two months. Cervix round, smooth, but hard, not voluminous, and directed backward. Applications on the 7th and 21st of September, and 9th of October. On this day the engorgement was much diminished, also the redness and anteversion. On the 18th the patient went out, feeling very well. State of the uterus on this day not indicated.

CASE V.—Engorgement of seven years' duration. Cervix voluminous, red, and presenting on its anterior lip, near the orifice, a small ulceration. Walking, impossible. Applications on the 24th of September and on the 9th and 20th of October. On the 23d of October the patient goes out. "The neck still seems a little swelled, in consequence of the application of the ointment." With the third application the engorgement had considerably diminished.

These are facts of evidently encouraging nature. In all cases the amelioration was obvious and rapid; and in spite of the reserved manner in which the author expresses the definitive result, the cure could have readily been perfected, at least for a time, in several cases. It is well to know that the application of the ointment produces, for some hours afterwards, more or less acute pain, which lasts from twelve to fourteen hours with intensity. According to M. Bernard, the pain seemed several times owing as much to the plugging of the vagina, and the compression of neighboring organs as to the action of the caustic. The whitish exudation, which was mentioned above, is generally formed at the end of five, six, or seven hours. The process of elimination, the cicatrization of the wound consecutive to the separation of the eschar, and the resolution of the concomitant inflammatory tumefaction requires about eight to ten days. It is, therefore, advisable generally to observe this interval between two applications.—(*Gaz. Hebdomad.*, May 21, 1858, from *Moniteur des Hôpitaux*, t. v., No. 140.)

VIII. *Thrombosis of the Cerebral Sinuses in Children.* By DR. GERHARDT, of Würzburg.

Thrombosis of the cerebral sinuses, in consequence of caries of the petrous portion of the temporal bone in adults, was first noticed by Lebert. He states, however, that of three hundred post-mortem examinations in children, he has not met with a single case of this disease. In ninety-six autopsies of children, Dr. Gerhardt observed seven cases of the disease, all of them occurring in infants less than six months old. Of the three cases, which he describes at length in his paper, we will present only one for the sake of illustration.

A foundling, eight weeks old, nourished artificially, suffered from a profuse diarrhœa, in consequence of which it was very much emaciated. Its skin was loose, and easily thrown into folds; dullness on the back of the chest on percussion, especially on its right side; consonant rales in some places; frequent laborious respiration; much cough; pulse and impulse of heart hardly perceptible; intense cyanosis, particularly of the face; highly distended vessels running from the large fontanelle to both temples. Murmurs of respiration and pulsation not perceptible at the head; pupils very much contracted; unconsciousness, amounting almost to lifelessness, interrupted only from time to time by cries and movements produced in the course of the examination. External jugular vein on the right side very little, on the left very much distended; distension increased on crying. No change in the symptoms until death.

*Autopsy.*—Slight atelectasis and limited hepatization of the lungs; tumefaction of the solitary follicles of the large intestines; membranes of the brain much reddened; the superior sinus longitudinal; right transverse sinus and several veins of the right hemisphere entering the former filled with thrombi, which were partly adherent, mostly hard, only in some places softened in their interior; brain soaked in effusion. From all the reported cases the following results may be inferred:—The disease is observed in children during the first three months of their lives; the subjects of it are badly nourished, and emaciated, complain of gastric disturbances, particularly diarrhœa; quick and intense collapse; feebleness; then failing of pulse, and cyanosis. The bones of the skull overlap each other, and the large fontanelle is sunken in. In the course of the disease, supervene loss of consciousness, disturbed innervation of the muscles of the eye, flexion of the neck, opisthotonos, rigidity of muscles, and death. The author sums up the diagnosis in the following:—

"If we observe in children violent cerebral symptoms following an acute catarrhal affection of the stomach and intestines, and then find that one of the jugular veins is collapsed, and the other highly dilated, both having had normal dimensions previous to the disease, and if this symptom presents itself with some distinctness and constancy, we are justified in diagnosticating thrombosis of the cerebral sinuses; and we will always find that side alone affected, or most so, upon which the vein is collapsed. We dare not, however, positively exclude the existence of thrombosis in case of absence of this symptom."

For the treatment of this disease the author recommends principally a good and proper nourishment. The recorded cases were treated chiefly with opium. As we see, however, from the foregoing, that thrombosis of the cerebral sinuses is observed generally in very young children, occurring together with diarrhœa, these observations, to which we draw the attention of practitioners, should serve as another warning in regard to the use of opium in early infancy, considering how easily it produces congestion of the brain.—(*Schmidt's Jahrbücher*, 1858, No. 4, from *Deutsche Klinik*, 1857, Nos. 45, 46.)

